# MagMap: A Parallel Decoding Scheme for Weak RFID Signals Using Middle State Points and Magnitude Extraction

**DOI:** 10.3390/s26123863

**Published:** 2026-06-17

**Authors:** Ruiqin Bai, Xiaopeng Zhang, Xiaoyu Lv

**Affiliations:** 1 College of Artificial Intelligence, Taiyuan University of Technology, Taiyuan 030024, China; bairuiqinty@163.com (R.B.); hebut_zxp@163.com (X.Z.); 2 Postdoctoral Workstation, China Railway 17th Bureau Group Co., Ltd., Taiyuan 030006, China; 3 North Automatic Control Technology Institute, Taiyuan 030006, China

**Keywords:** backscatter communication, parallel decoding, IQ domain clustering

## Abstract

As RFID systems become increasingly widespread, the limitations imposed by tag collisions on system performance are becoming more evident. Parallel decoding has attracted significant attention due to its ability to improve channel utilization and throughput. However, existing schemes often perform poorly when decoding weak signals. Several challenges remain, including the assumption of ideal channel conditions, difficulty in detecting tag state transitions, and the complexity of state cluster formations in the In-phase and Quadrature (IQ) domain. To address the above issues, this paper first experimentally verifies the ability of middle state points to segment tag states, and proposes a time-window-based pre-processing method to improve the density of state clusters in the IQ domain. Second, by leveraging the high vertical resolution of the reader, we propose an ideal magnitude calculation method and a matching strategy for combined state clusters under weak signal conditions. Finally, we propose MagMap, a parallel decoding scheme based on middle state points and magnitude extraction. Experimental results demonstrate that, under weak signal conditions, MagMap reduces the decoding BER (Bit Error Ratio) of received packets by more than 60% compared to the state-of-the-art.

## 1. Introduction

**Figure 1:**
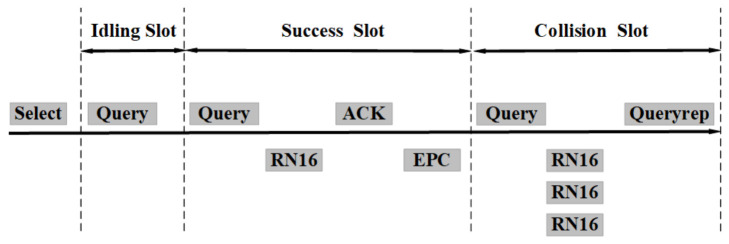
Three Response States in a Time-Slot.

RFID (Radio Frequency Identification) systems have been widely used in the fields of book management [1,2], physiological signal sensing, and trajectory identification [3,4,5] due to its tags’ passive, maintenance-free, and inexpensive characteristics. They adopt the Framed Slotted Aloha-based protocol as their communication standard. Therefore, tag collisions may occur when multiple RFID tags reply to the reader simultaneously. As shown in Figure 1, a frame contains multiple slots and the number of slots is defined by the reader before each communication. Each tag randomly selects a slot to communicate with the reader. When two or more tags select the same slot, tag collision occurs. The reader is unable to distinguish the colliding tags, resulting in communication failure. We refer to these slots as collision slots. Collision slots directly lead to a decrease in the throughput of the system. While increasing the number of slots reduces the collision probability, it also increases the number of empty slots, which lowers channel utilization. Therefore, enabling tags in collision slots to still communicate with the reader is the key to improve system throughput. To address this issue, two main approaches have been explored:

**Optimize transmission protocols.** Some studies [6,7,8,9] achieve parallel decoding by modifying existing communication protocols. For example, they change the Random number (RN) value returned by the tag in current protocols [10]. When tag collisions occur, the colliding tags can be distinguished by these modified RN values. In addition, there is a new parallel identification protocol designed for existing RFID systems. It can address the tag collision problem and improve the throughput of the system. However, these methods bring new problems. This requires designing new tags to implement the complex algorithms set by the protocol. As a result, it increases the overhead on the tag side.

**Improve the decoding method.** Studies [11,12,13,14,15] proposed to realize parallel decoding on the reader side, where energy is more abundant and computational performance is higher. Specifically, the cluster state in the IQ domain is identified based on the modulation or coding characteristics of the signal sent by the tag. Then, the signal of the tag is decoded according to the hopping of data points between cluster states. Studies [16,17] make use of multiple antennas and multiple frequencies to decode colliding packets from a large number of tags. Such schemes are able to decode the colliding signals that could not be handled under existing protocols. In this way, the throughput and channel utilization of the system can be improved.

While research on optimized transmission protocols may achieve parallel decoding, they may not be well-suited for the current protocol frameworks that are deployed and used on a large scale. At the same time, they increase tag-side overhead and are difficult to deploy at scale. In contrast, the approach of improving the decoding method places all computational overhead on the reader side. This makes it more practical to implement parallel decoding within the framework of existing protocols. Therefore, we adopt the latter approach in this work.

Parallel decoding on the reader side requires us to distinguish signals from different tags, which relies on accurate cluster classification and cluster state identification in the IQ domain. However, when facing weak received signals, existing parallel decoding schemes suffer from higher BER due to incorrect symbol clustering. This problem is particularly critical in practical deployments, where weak signals are common due to environmental factors and polarization mismatch between reader and tag antennas. As summarized in Table 1, representative prior works exhibit various limitations under weak signal conditions, such as poor scalability, sensitivity to superclustering, and reliance on stable cluster positions.

These limitations motivate us to design a new decoding scheme that can maintain robust performance even when received signals are weak. Building on this insight, we propose MagMap, a parallel decoding scheme based on preprocessing with middle state points and matching combined state cluster magnitudes. The core idea is to perform cluster state identification by matching the real magnitude of the combined state clusters in the IQ domain with the theoretical magnitude derived from subsequently extracted per-tag magnitudes. Additionally, we use data preprocessing and the reader’s high vertical resolution to amplify the weak magnitude differences caused by weak signals at the reader side.

In summary, our key contributions are twofold:

- **Pre-processing based on middle state points.** Designed to handle more complex clusters (e.g., overlapping clusters under the influence of noise, unstable cluster positions, and varying cluster densities) in the IQ domain. It improves the robustness and decoding accuracy of the system, particularly in the case of weak signals.
- **Magnitude matching algorithm.** Designed to identify all cluster states by leveraging the high vertical resolution at the reader side to distinguish the magnitudes of clusters, which reduces the complexity of cluster identification.

The remainder of this paper is organized as follows. Section 2 presents the background and motivation for our design. Section 3 details the design of MagMap. Section 4 evaluates MagMap and presents the experimental results. Section 5 concludes the paper.

## 2. Background and Motivation

To understand the behavior of collided tag signals under realistic conditions, we first examine the reader’s received signals based on data collected from our own experimental platform. All data presented in this section were collected from our USRP N210 reader with WISP tags based on the setup detailed in Section 4, ensuring that our analysis reflects real-world weak-signal scenarios.

**Figure 2:**
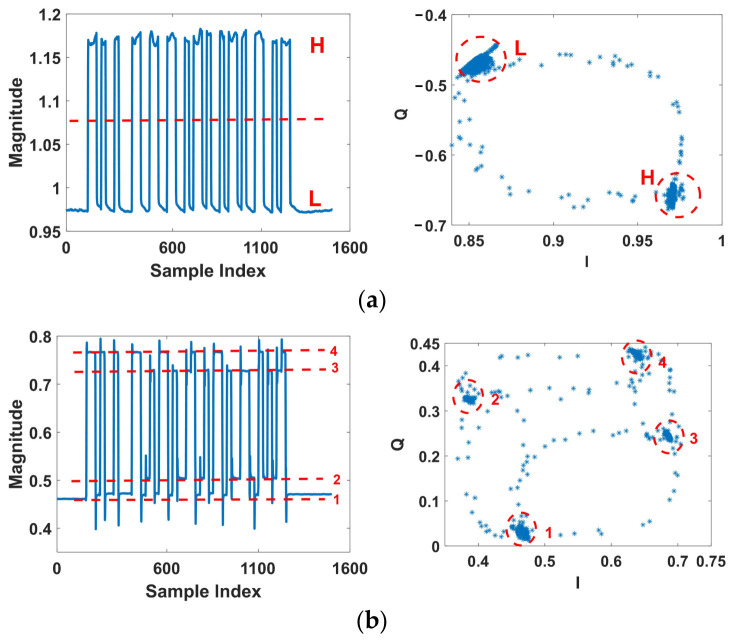
Received signals of different number of tags at the reader side: (**a**) signal of a tag in the time and IQ domains. (**b**) Signal of two tags in the time and IQ domains, the numbers 1–4 indicate the four clusters generated by the two tag signals in the IQ domains.

Passive RFID tags encode their data by reflecting or absorbing the carrier waves, thus resulting in two states: High (H) and Low (L). It generates two dense clusters which represent high and low states in the IQ domain, as shown in Figure 2a. The data points in the IQ domain contain magnitude and phase information. Specifically, the Euclidean distance from each data point to the origin corresponds to the magnitude information of that data point in the time domain. Therefore, the time-domain and IQ-domain representations are just different forms of the received data. When *N* tags are transmitting at the same time, their signals are superimposed at the reader side and the collided signals will have 2^N^ combined states, resulting in 2^N^ signal levels. A combined state is a theoretical linear combination of the states of all *N* tags, denoted as *S* = [*S*_1_, *S*_2_, …, *S_i_*, …, *S_N_*], where $S_{i}\in \left\{H,L\right\}$ indicates the state of tag *i*, which can be either H or L. As shown in Figure 2, when only one tag is present, we can decode its signal using a simple threshold-based division. However, when multiple tags collide, this straightforward approach no longer works. Fortunately, the combined state of the collided tags can correspond to distinguishable dense clusters in the IQ domain. Take the example of two colliding tags, the resulting four combined states can be denoted as LL, HL, LH, HH. After identifying the combined state of the clusters in the IQ domain, we can decode the signal of each colliding tag based on the hopping of data points between clusters.

There are several recent schemes to realize parallel decoding based on the above idea. BiGroup [14] realizes parallel decoding by extracting the bit boundaries of each tag in the time domain and corresponding to the clusters in the IQ domain. FlipTracer [15] achieves parallel decoding by leveraging the different transition probabilities between combined state clusters in the IQ domain.

**Figure 3:**
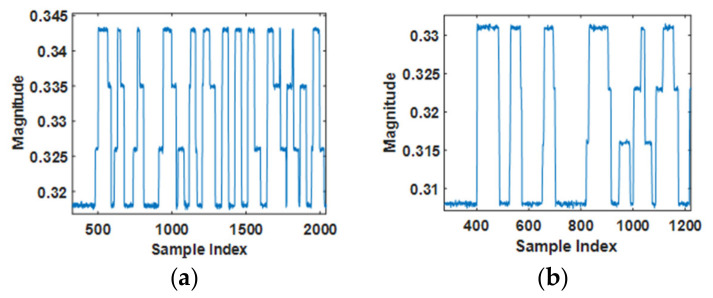
Unsynchronized tag signals at the reader side: (**a**) Different response delay. (**b**) Unstable bit duration.

**Figure 4:**
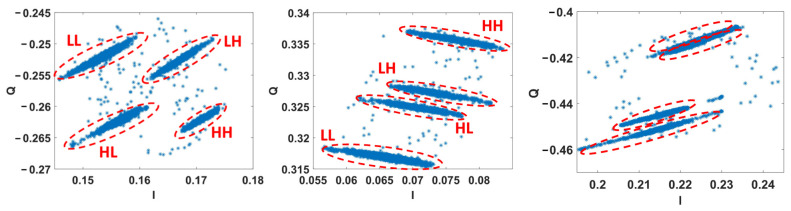
Possible different states of clusters in the IQ domain.

Although existing schemes can support parallel decoding, they still face the following problems:

(1) Poor scalability under weak received signals. During communication between the reader and tags, the received signal strength at the reader side varies significantly due to factors such as location, antenna polarization, and environmental conditions. In some cases, the signal strength difference can be as large as an order of magnitude. When facing weak received signals or an increasing number of colliding tags, existing schemes fail to perform reliable symbol clustering, which subsequently affects cluster identification and decoding.
(2) Unsynchronized Collided Signals. Due to variations in response delay and bit duration (i.e., symbol period) among tags—factors largely determined by the tag’s clock rate, manufacturing process, and physical location—the collided signals are often unsynchronized. This leads to misalignment at the reader side, as illustrated in Figure 3. Figure 3a shows unsynchronized tag signals caused by different response delays, while Figure 3b shows unsynchronized signals caused by unstable bit durations.
(3) Instability in the IQ Domain. Theoretically, the channel coefficients of the colliding tags are stable and the collided signal is the linear combination of these tags’ channel coefficients. Thus, the collided signals with specific combined states will exhibit specific positions in the IQ domain [12]. However, in practice, the positions of clusters in the IQ domain are not stable, and clusters may overlap due to noise. Figure 4 illustrates the instability of the clusters in the IQ domain under different noise levels. The left subplot (SNR = 25 dB) shows well-separated clusters representing an ideal case with minimal noise. The middle subplot (SNR = 15 dB) shows clusters that begin to spread, with boundaries becoming less distinct. The right subplot (SNR = 8 dB) shows clusters that heavily overlap, exhibiting superclustering due to severe noise and surrounding interference.

Moreover, under the influence of noise, both the density of each cluster and the distances between clusters become uncertain. As a result, superclustering—a phenomenon in which clusters are close to or even overlap with one another—is likely to occur in practice. Density-based symbol clustering methods often fail when superclustering appears, as they cannot separate the overlapping clusters within superclusters.

To handle superclusters, existing schemes need to exploit their internal complex correlations. For instance, Canon [19] attempts to solve the overlapping cluster problem by exploring multi-channel information, assuming that state clusters overlapping on one channel may not overlap on another channel. Hubble [18] proposes a scheme to identify the combined states of collided signals using Pearson’s Chi-Square test.

To address the above problems, we take advantage of the high vertical resolution provided by modern RFID readers. In the case of weak signals, we achieve cluster state identification by exploiting the magnitude differences produced by the superposition of different tags. In the face of unsynchronized tag signals and IQ domain instability, we leverage these phenomena to extract the magnitude of individual tags while avoiding the negative impact of cluster position variations on cluster state identification.

**Figure 5:**
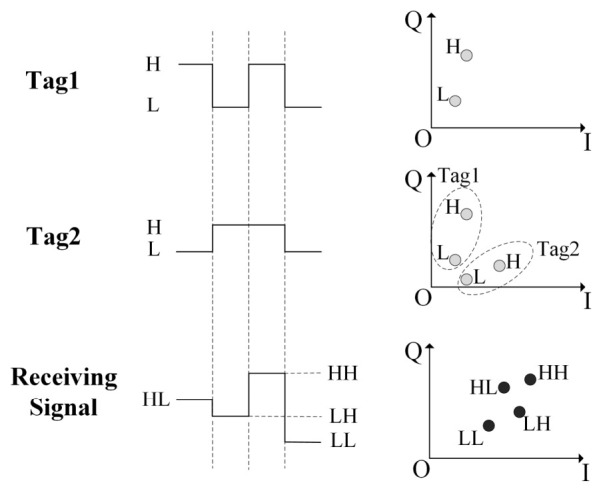
Theoretically two signals add up at the reader side.

Figure 5 illustrates the theoretical generation of signals in the time domain and IQ domain when two tags collide. We find that when two tags collide, their signals add up at the receiver. Consequently, if the magnitude of tag 1 is greater than tag 2, then it means that the combined state HL has a greater magnitude than LH. Based on this finding, we calculate the theoretical magnitude of each combined state after extracting the magnitude of each tag. Next, we extract the real magnitude of each combined state cluster in the IQ domain. Finally, we complete the cluster state identification for parallel decoding by matching the theoretical magnitudes with the real magnitudes.

It is worth noting that the main reasons for performing single-tag magnitude extraction and real magnitude extraction of state clusters in the IQ domain are as follows:

(1) The distance from the cluster to the origin in the IQ domain corresponds to the magnitude level in the time domain. Moreover, the clusters in the IQ domain contain the phase information of the signal. It can help us to distinguish the clusters better. All the data points of the same state are aggregated together at the same time. We can do some processing like calculating the average or other operations.
(2) The magnitude information in the time domain is more dispersed. We are not able to extract the magnitude information by dividing the threshold. However, the data in the IQ domain is naturally categorized after clustering. It is convenient for us to extract the magnitude information accurately.

## 3. Design

**Figure 6:**
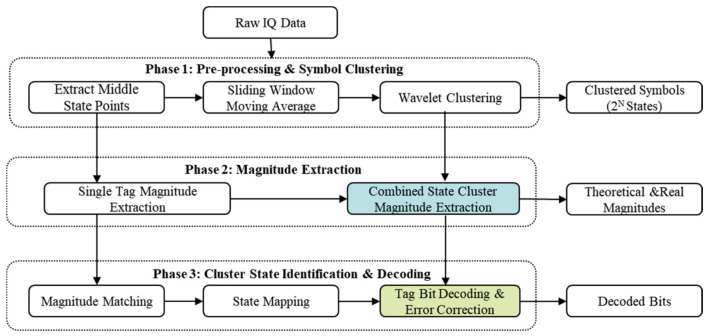
The workflow of MagMap.

Figure 6 illustrates the workflow of MagMap. The proposed design consists of three main phases. Phase 1 performs pre-processing and symbol clustering. Phase 2 extracts both single-tag magnitudes and combined-state cluster magnitudes. Phase 3 conducts cluster state identification and decoding. To obtain the complete set of theoretical cluster states, we must ensure that all possible combinations of tag signals are captured at the reader side. Our current strategy is to initiate pre-processing and symbol clustering only after receiving the complete data packet, thereby guaranteeing the identification of all 2*^N^* cluster states, where *N* is the number of colliding tags.

### 3.1. Pre-Processing and Symbol Clustering

The purpose of data pre-processing is twofold: (1) to smooth the received data so that the combined state clusters in the IQ domain become denser and the magnitude differences between clusters become more distinct; (2) to improve system’s noise resistance, particularly under weak signal conditions.

The number of samples within each bit duration at the reader side depends on the reader’s sampling rate and the tag’s backscatter link frequency (BLF), which is determined by the parameters sent by the reader. Therefore, the number of samples per bit duration can be theoretically determined in advance for each tag’s transmission. However, due to the unsynchronized responses of tags, the bit duration of each tag is unstable. To achieve the above pre-processing goals, we use a sliding window to perform moving average smoothing. The key challenge lies in setting the window length. If the window length is too large, the window may include data points from neighboring states, which significantly affects the smoothing process and reduces the magnitude differences between different signal levels.

**Figure 7:**
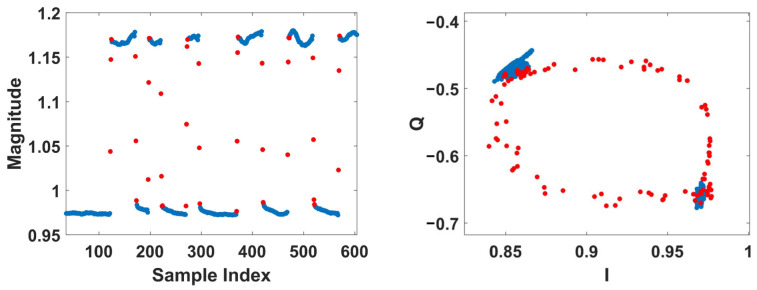
Middle state points in the time domain and IQ domain (Red highlighted points).

At the same time, we observed that due to the internal hardware limitations of the tags, the transition between tag states is not instantaneous. Specifically, there exist data points between state transitions. As shown in Figure 7, we refer to these as middle state points (MPs). These points are clearly observable in the time domain as sampling points between states. In the IQ domain, MPs appear as discrete data points located between dense clusters. MPs exist precisely at the moments when state transitions occur. Therefore, the distance (in terms of data point count) between different MPs corresponds to the bit duration of a particular combined state.

**Figure 8:**
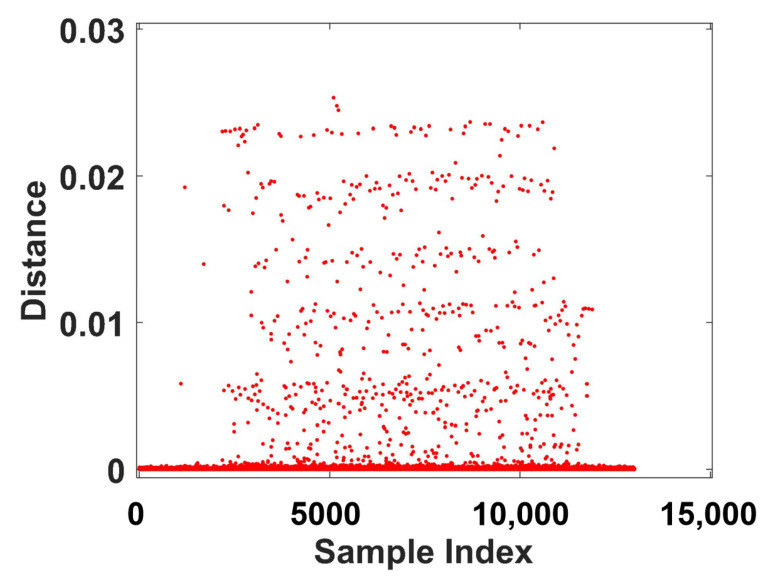
Euclidean distance to the last adjacent data point.

In summary, we propose a sliding window data pre-processing method based on middle state points. We first determine the window length based on the MPs and then apply the sliding window for data pre-processing. The process of determining the window length is as follows:

**Extract the middle state points.** Since MPs may deviate from the original states, we calculate the Euclidean distance between each data point and its immediate preceding data point to obtain the deviation. As shown in Figure 8, most data points are concentrated at the bottom, while points that are far from their previous data point are identified as MPs. We set a threshold to extract the MPs. This threshold is defined as the average value of the carrier wave before the signal arrives, representing the current noise floor of the channel. In practice, although threshold-based extraction may occasionally select an in-state point as an MP due to noise fluctuations, this does not affect subsequent processing—it merely results in a slightly smaller sliding window.

**Figure 9:**
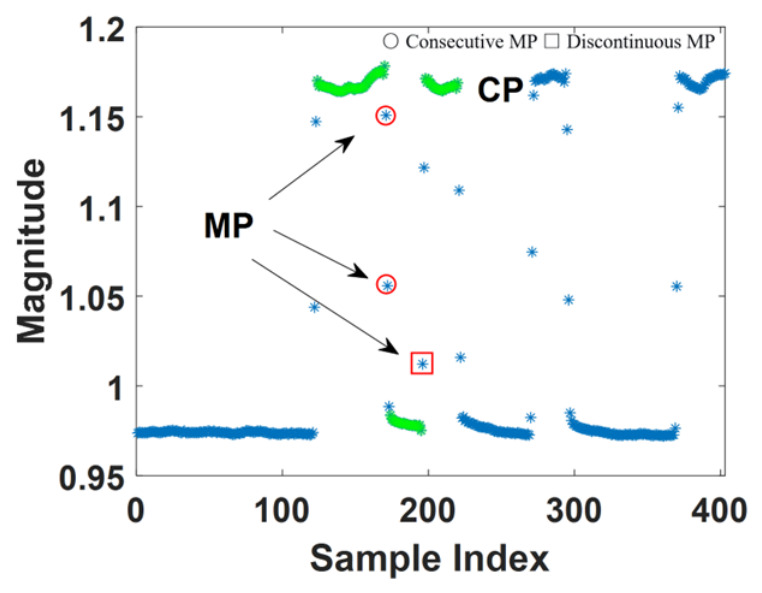
Processing middle state points. The figure legend shows the following: MP (middle state points), CP (categorized points), consecutive MP (circles, continuously sampled during transition), and discontinuous MP (squares, marking state boundaries). The length of a discontinuous MP segment determines the window length, the asterisk * denotes the identified MP.

**Process the middle state points.** In contrast to MPs, the remaining data points belong to categorized points (CPs), i.e., data points that belong to a combined state cluster. As shown in Figure 9, the two states and the MPs are clearly distinguishable. The two states and the MPs are clearly distinguishable. We need to identify consecutive and discontinuous middle state points. The consecutive MPs (indicated by circles in the figure) are continuously sampled during a state transition and require no action. The discontinuous MPs (indicated by squares) mark the boundaries between states: the gap (number of data points) between a square and the previous circle corresponds to the duration of a certain combined state and serves as our sliding window length.

**Figure 10:**
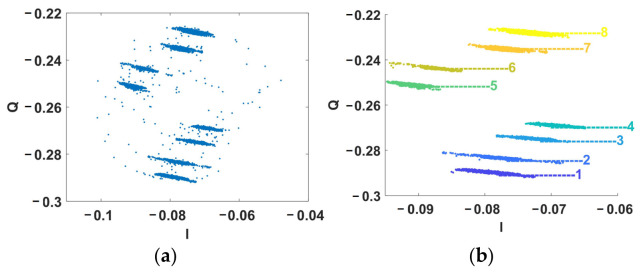
Symbol clustering: (**a**) Before clustering; (**b**) After clustering with wavelet clustering [20]. The numbers 1–8 indicate combined state clusters, distinguished by different colors.

After pre-processing the signals to reduce noise and improve clarity, we use a clustering algorithm to distinguish different states in the IQ domain. Clustering groups similar signal states together, thereby reducing decoding complexity. Existing approaches [13,14] use grid-based processing for faster clustering, but this can lead to scalability issues because the number of grids strongly influences computational complexity. To address this, we use wavelet clustering [20], which is also grid-based but offers advantages in both efficiency and scalability, especially under weak signal conditions where higher resolution is needed without sacrificing efficiency. Once clustering is complete, we obtain the classification of each combined state cluster and its corresponding cluster number, as shown in Figure 10.

### 3.2. Single-Tag Magnitude Extraction and Combined State Cluster Magnitude Extraction

The magnitude differences between combined state clusters in the IQ domain become more pronounced after pre-processing. We leverage the response asynchrony of tags to extract the magnitude of a single tag. Due to instability in tags’ internal clocks, tag asynchrony is a common issue. For example, work [21] discusses the problem of asynchronous tag reception, while study [14] detects bit boundaries of each tag by exploiting tag misalignment and asynchrony. Although asynchrony may pose challenges for some parallel decoding methods, it is possible for us to take full advantage of it. Just as BiGroup [14] can extract the start position of each tag, we can also extract the magnitude of each tag by detecting its starting position.

**Figure 11:**
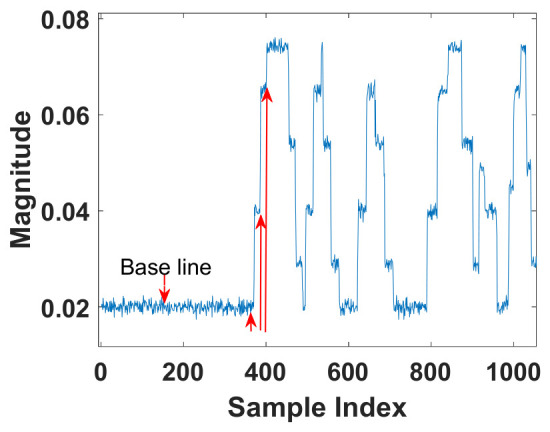
Tags join the transmission at different times.

To facilitate understanding of our method for extracting single-tag magnitudes, we describe the process in the time domain. First, a known preamble is transmitted before the payload signal, which is also part of the information sent by the tag. Then, when a new tag joins the communication, the tags that have already joined remain in the high state. Figure 11 illustrates the starting points when different tags join the communication. The first arrow indicates the state when no tag is transmitting, while the following three arrows indicate the moments when three different tags join the transmission. Due to the arrival of new tags, the combined state changes accordingly. We take the full low state (i.e., no tag transmitting) as the base state and obtain the magnitude of each tag based on the state transition caused by the newly arriving tag. The magnitude of a single tag is given by Equation (1):

(1) $$a_{i}{=A}_{i}-A_{p},\;i=1,\;2,\;3,\;\ldots ,\;N$$

where $a_{i}$ denotes the magnitude of tag *i*, *A_i_* denotes the cluster state magnitude after the tag *i* joins the transmission, *A_p_* denotes the previous combined state magnitude, and *N* is the number of collided tags.

Once the magnitude of each tag is extracted, we can calculate the theoretical magnitude of each possible combined state. There are 2^N^ combined states, each corresponding to a unique combination of tag states. Let $S_{i,k}$ indicate the state of tag *i* in the *k*-th combination, where 0 represents L and 1 represents H. The theoretical magnitude of the *k*-th combined state is given by (2):

(2) $$M_{k}\;=\sum_{i=1}^{N}(S_{i,k}\cdot \;a_{i}),\;k=1,2,3,\ldots {,2}^{N}$$

After clustering, each combined state is assigned a unique cluster number. We extract the magnitude of each combined state cluster in the IQ domain as the real magnitude value. Let *C_k_* be the number of data points in the *k*-th cluster, and let *p_j_* be the magnitude of the *j*-th point in that cluster (i.e., its Euclidean distance to the origin). The real magnitude of the *k*-th combined state is given by Equation (3):

(3) $$R_{k}\;=\frac{1}{C_{k}}\sum_{m=1}^{C_{k}}p_{j},\;k=1,2,3,\ldots {,2}^{N}$$

The proposed magnitude extraction method relies on the natural asynchrony of tag responses. In the extreme case where two tags are almost perfectly synchronized, the detection of distinct starting positions becomes challenging. However, such perfect synchronization is highly unlikely in practice due to inherent variations in tag clock sources, manufacturing tolerances, and propagation delays. Even if temporal separation is significantly reduced, the magnitude information can still be extracted from the combined state transitions (e.g., from low state to HL or LH) as long as there is any measurable misalignment. Even a small temporal offset between tag responses—which is almost always present due to inherent variations in tag clock sources, manufacturing tolerances, and propagation delays—is sufficient for reliable magnitude extraction.

### 3.3. Cluster State Identification and Decoding

After the magnitude extraction, we get the theoretical magnitude and the real magnitude. Each combined state cluster can then be identified by matching these two sets of magnitudes.

**Figure 12:**
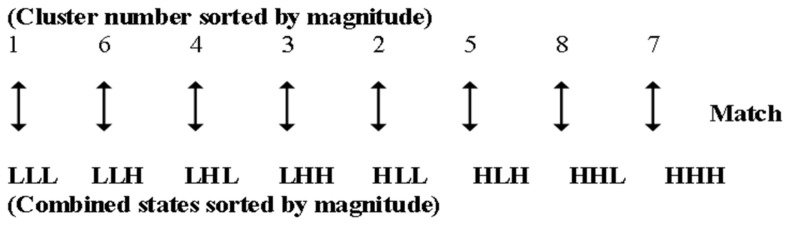
Cluster state identification diagram.

**Figure 13:**
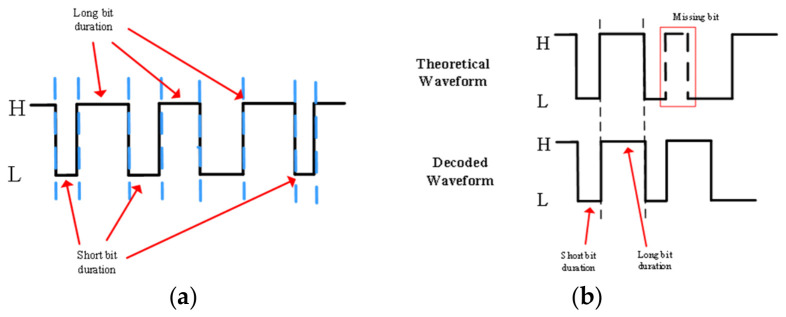
Decoding problems: (**a**) Unstable bit duration (**b**) Missing bits.

As shown in Figure 12 (with the identified clusters corresponding to those in Figure 10), cluster state identification is completed. We can then output the sequence of high and low states (i.e., 0 1 sequence) of each colliding tag for decoding. However, several challenges remain in decoding:

**(1)**  **Unstable bit duration.** The bit duration of the tag signal is unstable due to the instability of the tag’s internal clock. In addition, coupled with the effect of noise, the output high and low state sequence is not a completely stable signal. The duration of each bit has a long or short duration. As shown in Figure 13a, the long bit duration represents two bits and the short bit duration represents one bit. It is obvious that the duration is not stable.
**(2)**  **Missing bits.** The message sent by the tag will be encoded by FM0 or Miller [10], which follows a specific format. For example, message 0 is represented by “01” or “10”, and message 1 is represented by “11” or “00”. For message 0, 0 and 1 exist in pairs. As shown in Figure 13b, what appears to be a normal waveform actually contains missing bits, which ultimately leads to a high BER.

**Figure 14:**
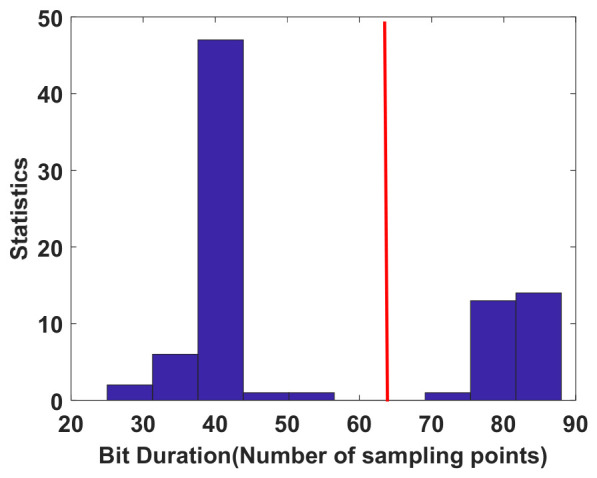
Statistical analysis of bit durations. The dividing line between long and short bit durations is determined based on the statistical results.

For these issues, a traditional single decoder would require extracting bit boundaries to detect possible errors. However, the instability of bit duration leads to cumulative errors in boundary extraction. Therefore, we need to analyze the encoding format to find a more accurate decoding method. To address unstable bit duration, we design a decoder based on statistical analysis. After outputting the high/low state sequence, we statistically analyze the lengths of long and short bit durations. As shown in Figure 14, a dividing line between long and short bit durations is determined based on the statistical results. The bit sequence is then determined according to this dividing line.

To address the missing bit issue, we rely on encoding characteristics. Short bit durations typically appear in pairs. Therefore, when we observe a single short bit duration in isolation (as shown in Figure 13b), we can infer that a bit is missing. Subsequently, we perform timely error correction.

## 4. Implementation and Evaluation

**Figure 15:**
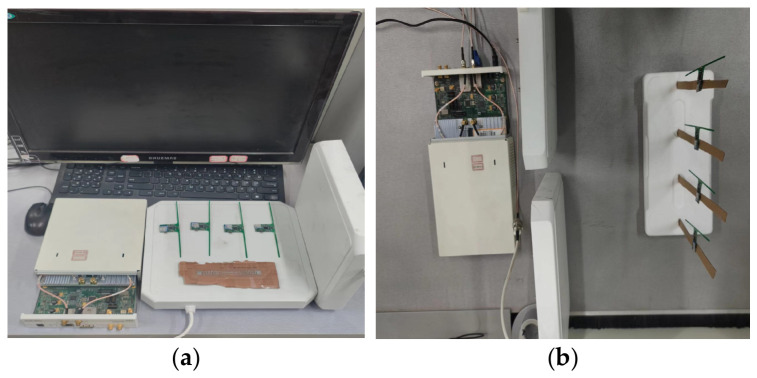
The hardware platform: (**a**) Experimental equipment. (**b**) Experimental setup.

The hardware platform is shown in Figure 15. Figure 15a shows a static display of the experimental equipment. Figure 15b shows the relationship between the experimental equipment during the experiment. MagMap is built based on the USRP N210 software-defined radio platform (Ettus Research LLC, Santa Clara, CA, USA) with UBX RF daughter-boards and two 900 MHz circular antennas. The USRP N210 can be configured with various daughterboards, including the UBX RF daughterboards, which provide access to a wide range of frequency bands.

Reader side: Because of the encapsulated nature of commercial readers, we only have access to some upper-level information, such as tag ID, phase, RSSI (received signal strength), etc. But we cannot obtain the physical layer information sent by the tag. Therefore, we use USRP N210 software radio reader with UBX RF daughter-boards as the reader. The default sampling rate and Tx-Gain of the antenna are set to 20 MHz and 20 dB, respectively.

Tag side: We use the Wireless Identification and Sensing Platform (WISP) as the tag. Each tag sends a message consisting of a 6-bit known preamble, 96 message bits, and 1 known dummy bit. The packet length is 206 bits after FM0 encoding. The bit rate is 125 kbps.

We compare MagMap with FlipTracer [15]. FlipTracer is the most relevant and competitive baseline for weak-signal parallel decoding: it is published in IEEE/ACM ToN, specifically designed for low-SNR scenarios, and reportedly outperforms other existing methods under weak signal conditions. Therefore, it serves as the strongest available baseline to evaluate MagMap. First, we evaluate the effect of our preprocessing in the time and IQ domains. Then, we compare the performance of the two schemes in terms of both Bit-Error-Rate (BER) and throughput. In the experiment, we vary the number of tags from two to four and adjust the gain of the transmitting antenna to change the SNR of the signals. Specifically, we set the SNR to 15 dB, 20 dB, and 25 dB, and receive 40 collided packets for each setting. We then use the two different approaches (MagMap and FlipTracer) to decode the information of the tags.

### 4.1. Pre-Processing

**Figure 16:**
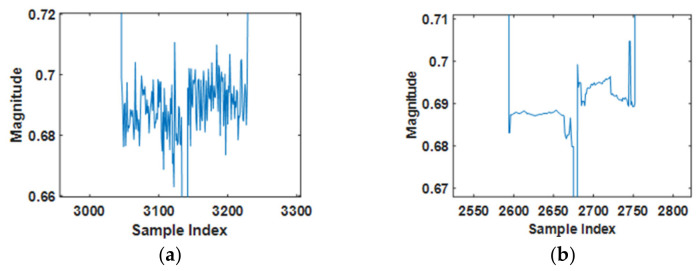
Change in close magnitude. (**a**) Before pre-processing. (**b**) After pre-processing.

As shown in Figure 16, we compared the effects before and after pre-processing in the time domain and IQ domain. Figure 16a shows the two states before pre-processing in the time domain. When the number of tags increases, it may yield two combined states with very similar magnitudes, even if the difference in magnitude of each tag is large. It looks like the magnitude differences are no longer distinguishable, but there is still a relative magnitude difference. Figure 16b shows the two states after pre-processing. We can see that the magnitude is already clearly distinguishable in the time domain. Then it also makes a significant difference in magnitude between the two corresponding states in the IQ domain. The difference in the horizontal axis is that the pre-processing removes some discrete points between states and does not affect the code elements.

**Figure 17:**
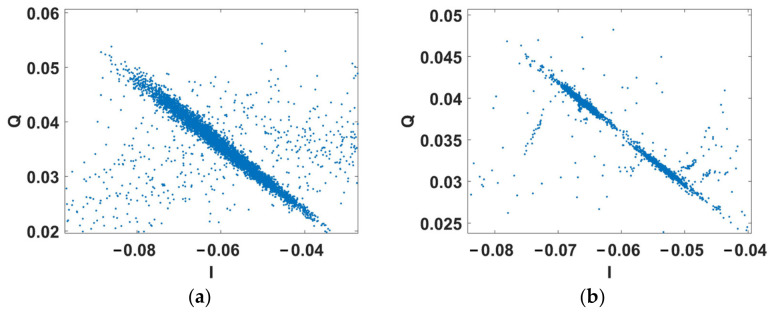
Change in overlapping clusters. (**a**) Before pre-processing. (**b**) After pre-processing.

Figure 17 shows the effect of the two combined state clusters before and after pre-processing in the IQ domain. As shown in Figure 17a, the two state clusters are no longer distinguishable due to the noise. The reason for this is that the distance between the two overlapping clusters is small. Meanwhile, the density of clusters is lower under the influence of noise. Then the two cluster boundaries overlap and it looks like one cluster. However, even if the two clusters overlap, there is still a relatively different cluster center. Figure 17b shows the two distinguishable combined state clusters after pre-processing. When data points in the same state shrink toward their own cluster centers, the overlapping edges separate. Therefore, it is robust to cluster the overlap caused by noise after pre-processing.

### 4.2. BER

**Figure 18:**
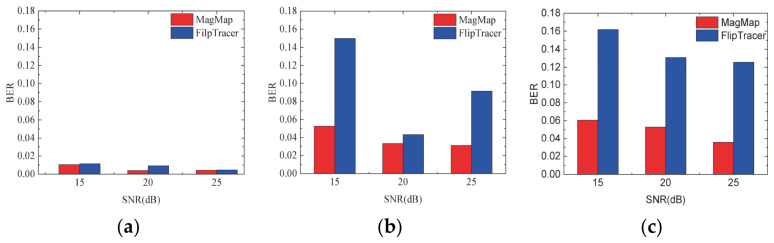
Overall BER versus SNR for different numbers of colliding tags: (**a**) Two tags. (**b**) Three tags. (**c**) Four tags.

Figure 18 illustrates the overall BER for different numbers of tags across varying SNRs. When a collision occurs, the decoding scheme may generate multiple candidate packets. To evaluate decoding performance, we compare each candidate packet with the original transmitted packets and record the minimum BER. It is evident that MagMap outperforms FlipTracer in decoding. When few tags collide (e.g., two tags), both schemes maintain a relatively low BER, staying below 0.012. However, as the number of tags increases, the performance gap between the two schemes widens. Specifically, when the SNR is 15 dB (indicating weak received signal strength), MagMap still achieves a BER below 0.06. Compared to FlipTracer, MagMap reduces the BER by 65% and 62% in the cases of three and four collided tags, respectively. This improvement is attributed to the data preprocessing stage, which becomes increasingly effective as the number of tags rises and the received signal weakens.

Furthermore, while MagMap’s BER consistently decreases with increasing SNR, FlipTracer’s BER occasionally increases. Upon examining each stage of the process, we attribute this anomaly to potential inaccuracies in cluster classification and lost bits during decoding. These issues can lead to accumulated errors. Consequently, a high BER in a particular data packet can disproportionately affect the overall system performance. Indeed, in our subsequent experiments, we frequently observed that a single packet with a high BER often resulted in a higher overall BER.

**Figure 19:**
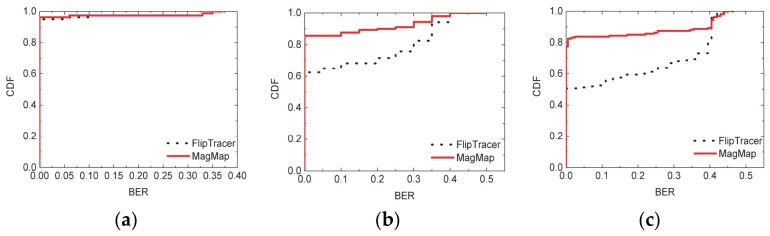
CDF of BER for different numbers of colliding tags under weak signal (SNR = 15 dB): (**a**) Two tags. (**b**) Three tags. (**c**) Four tags.

While the average BER shown in Figure 18 reflects the general decoding capability for collided tags, individual high BER events can substantially impact the overall performance in practice. To better characterize this, Figure 19 presents the CDF (Cumulative Distribution Function) of BER when the received signal is weak (SNR = 15 dB). For collisions involving three and four tags, MagMap achieves 0-bit error in 80% and 77% of collisions, respectively, compared to only about 60% and 50% for FlipTracer. Moreover, the probability of an unacceptable BER (BER > 0.3) in MagMap is 62% lower than that in FlipTracer. These results confirm that MagMap achieves a lower BER because it fully leverages the advantages of preprocessing and decoding, tolerating the complexity in the IQ domain even under weak received signal conditions.

### 4.3. Throughput

**Figure 20:**
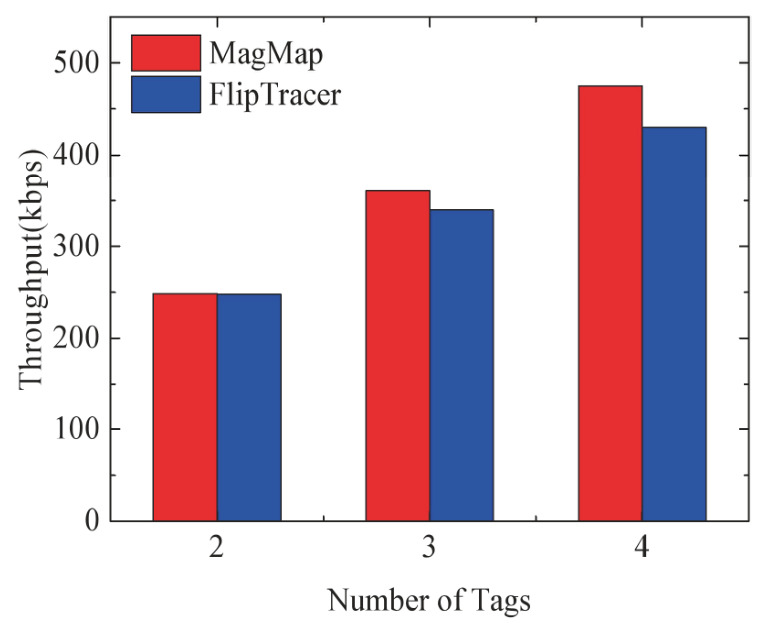
Throughput comparison of different schemes at SNR = 15 dB.

Figure 20 illustrates the throughput of different schemes at an SNR of 15 dB. Throughput, defined as the number of correctly transmitted bits per unit time, is directly affected by the BER; a higher BER reduces the system throughput. Theoretically, the maximum throughput achieved by parallel decoding equals the sum of the bit rates of all concurrent tags. For example, with two concurrent tags each operating at 125 kbps, the theoretical maximum throughput is 250 kbps.

The results show that MagMap achieves throughput close to the theoretical maximum, outperforming FlipTracer. When two tags collide, the throughput difference between the two schemes is marginal, with MagMap outperforming FlipTracer by 0.2%. However, when three or four tags collide, MagMap’s throughput exceeds that of FlipTracer by 11% and 12%, respectively.

### 4.4. Complexity and Scalability Analysis

We briefly analyze the computational complexity of each step in MagMap and compare it with FlipTracer.

Preprocessing (middle state point extraction and sliding-window smoothing): Both operations are linear in the number of IQ samples, i.e., *O*(*L*), where *L* is the total number of samples.

Symbol clustering: We use wavelet clustering, which has an average complexity of *O*(*L logL*).

Magnitude extraction and matching: This step involves computing *2^N^* cluster magnitudes (where *N* is the number of colliding tags) and comparing them with theoretical values, resulting in *O*(*2^N^*) operations.

Overall, the dominant cost is the wavelet clustering step. FlipTracer similarly relies on clustering and transition probability calculations, with comparable complexity. MagMap introduces a modest overhead due to the additional preprocessing, but this is justified by the substantial BER reduction achieved under weak signal conditions.

Regarding scalability, we considered up to four colliding tags in our experiments. This range is representative of typical collision scenarios in practical RFID systems, where most anti-collision protocols (e.g., *Q*-algorithm in EPC C1G2) limit the expected number of colliding tags per slot to a small constant. Moreover, as the number of tags *N* increases, the number of combined states grows exponentially as *2^N^*, which could increase the complexity of clustering and magnitude matching. However, for *N* ≤ 6, the clustering step remains computationally feasible *O*(*L logL*) with *L* ≈ 4000 samples). Beyond *N* = 6, the separation between combined state clusters may become less distinct under weak signal conditions, potentially affecting decoding accuracy. We leave further scalability improvements (e.g., hierarchical clustering or compressive sensing) for future work.

## 5. Conclusions

We have proposed MagMap, a parallel decoding scheme designed for weak collided signals. By exploring the physical-layer nature of collided tag reception—specifically, signal superposition—and leveraging the reader’s high vertical resolution, MagMap achieves robust cluster state identification and decoding. Furthermore, our data preprocessing effectively handles complex clusters in the IQ domain under weak signal conditions. Experimental results demonstrate that the proposed scheme reduces the bit error rate (BER) by more than 60% in weak received signal scenarios.

## Figures and Tables

**Table 1:** Comparison of existing parallel decoding schemes and their limitations relative to MagMap.

Method	Category	Key Idea	Limitations
DDC [6]	Protocol-based	Modify RN values to distinguish colliding tags	Requires tag-side changes; not backward compatible with deployed RFID systems
PIP [7]	Protocol-based	Parallel identification protocol	High tag overhead; increases tag complexity; difficult to scale
TagTone [8]	Protocol-based	Multi-frequency analysis for scalable RFID	Needs additional frequency resources; tag-side complexity
BiGroup [14]	Decoding-based	Extract bit boundaries from time domain	Unstable bit boundaries under weak signals
FlipTracer [15]	Decoding-based	Use transition probabilities between clusters	Performance degrades significantly at low SNR
Hubble [18]	Decoding-based	Pearson’s Chi-square test for overlapping clusters	Complex and high latency
MagMap (Ours)	Decoding-based	Middle state points and matching combined state cluster magnitudes	—

## Data Availability

The data presented in this study are available on request from the corresponding author. The data are not publicly available due to privacy restrictions.

## Abbreviations

The following abbreviations are used in this manuscript:

BLF	Backscatter Link Frequency
MP	Middle State Point
RFID	Radio Frequency Identification
RN	Random Number
IQ	In-phase and Quadrature
